# Editorial: The etiology and pathogenesis of craniomaxillofacial birth defects

**DOI:** 10.3389/fdmed.2025.1592762

**Published:** 2025-03-28

**Authors:** Huaxiang Zhao, Wenbin Huang, Huan Liu, Yongchu Pan

**Affiliations:** ^1^ Key Laboratory of Shaanxi Province for Craniofacial Precision Medicine Research, College of Stomatology, Xi'an Jiaotong University, Xi'an, China; ^2^ Department of Orthodontics, Guangdong Provincial High-level Clinical Key Specialty, Guangdong Province Engineering Research Center of Oral Disease Diagnosis and Treatment, Shenzhen Clinical Research Center for Oral Diseases, Stomatological Center, Peking University Shenzhen Hospital, Shenzhen, China; ^3^ State Key Laboratory of Oral & Maxillofacial Reconstruction and Regeneration, Key Laboratory of Oral Biomedicine Ministry of Education, Hubei Key Laboratory of Stomatology, School & Hospital of Stomatology, Wuhan University, Wuhan, Hubei, China; ^4^ Frontier Science Center for Immunology and Metabolism, Wuhan University, Wuhan, Hubei, China; ^5^ TaiKang Center for Life and Medical Sciences, Wuhan University, Wuhan, Hubei, China; ^6^ State Key Laboratory Cultivation Base of Research, Prevention and Treatment for Oral Diseases, Nanjing, China; ^7^ Jiangsu Province Engineering Research Center of Stomatological Translational Medicine, Nanjing, China; ^8^ Department of Orthodontics, The Affiliated Stomatological Hospital of Nanjing Medical University, Nanjing, China

**Keywords:** craniofacial defect, craniofacial development, next-generating sequencing, orofacial cleft (OFC), etiology and pathogenesis

**Editoral on the Research Topic**
The etiology and pathogenesis of craniomaxillofacial birth defects

Craniomaxillofacial birth defects, including orofacial clefts, craniosynostosis, ocular anomalies, and malformations of the nose and ears, account for one-third of all congenital defects. These defects not only affect facial appearance but also disrupt craniofacial and oral function, posing significant risks to newborn survival and contributing to long-term complications (1).

The formation of the human skull and face is a highly intricate morphogenetic process involving precisely orchestrated cellular and molecular events. Disruptions in these processes, whether environmental or genetic, can lead to craniofacial anomalies. In recent years, advancements in high-throughput sequencing and gene-editing technologies have led to the identification of numerous causative genes and a deeper understanding of their pathogenic mechanisms (2–4). This Research Topic comprises seven articles that explore the etiology of several craniofacial birth defects and discuss the application of emerging technologies in the early diagnosis of these anomalies.

Although high-throughput sequencing is widely used, the appropriate design of testing strategies remains critical for the early molecular diagnosis of patients. Lai et al*.* found that exome sequencing in trios achieved a significantly higher diagnostic yield than in singletons among individuals suspected of genetic disorders. They also observed that structural anomalies, such as global developmental delay, had a higher diagnostic rate than functional abnormalities like muscular hypotonia. Additionally, inheritance patterns played a key role in diagnostic success. This study underscored the effectiveness of exome sequencing in early diagnosis and highlighted essential factors to consider, including testing strategies, disease types, and inheritance patterns.

The next two studies focus on two of the most prevalent craniofacial anomalies: orofacial clefts and craniosynostosis. Yan et al*.* performed exome sequencing on 107 singleton pregnancies diagnosed with fetal orofacial clefts and their parents, identifying clinically significant variants in 11.2% of cases. Regarding craniosynostosis, Topa et al*.* conducted genome or exome sequencing in a cohort of 59 patients who had previously undergone targeted analysis without identifying causal variants. They found that 38% of syndromic craniosynostosis cases had a genetic cause, and many potentially relevant variants were detected in the majority of the remaining families without prior causal findings. These results reinforce the value of genome and exome sequencing as powerful diagnostic tools for craniosynostosis. In addition, Topa et al*.* highlighted the role of human phenotype ontology-term-driven variant filtration in identifying novel candidate genes/variants associated with craniosynostosis.

Once potential pathogenic variants are identified, functional assays *in vitro* and/or *in vivo* are often required to validate their pathogenicity. In this issue, Zhao et al*.* demonstrated that two novel variants impair the function of FOXL2, a known causal gene for blepharophimosis, ptosis, and epicanthus inversus syndrome, providing valuable insights into the genetic basis of this diseases.

In recent years, the expansion of sequencing studies has significantly enriched disease-related databases, enabling data mining and re-analysis as effective approaches for studying craniofacial anomalies. Wang et al*.* utilized open datasets to identify TFE3 and TP53 as novel biomarkers for chronic rhinosinusitis with nasal polyps by analyzing differentially expressed genes. These findings not only enhance our understanding of the molecular mechanisms underlying the disease but also provide potential targets for future therapeutic strategies.

Case reports continue to be a valuable source of information for understanding the genetic basis of craniomaxillofacial anomalies. Wu et al*.* described a case of Silver–Russell syndrome with an 8q12 deletion including the *PLAG1* gene, accompanied by a literature review. Meanwhile, Xu et al*.* reported a case of complete trisomy 9 with an unusual phenotypic presentation and reviewed the clinical features of fetuses affected by this chromosomal abnormality.

Overall, this Research Topic highlights the application of advanced technologies in the study of the etiology and pathogenesis of craniomaxillofacial birth defects. It also emphasizes research strategies and the potential for broader application in the future. We hope this collection of studies provides valuable insights to researchers, extending beyond those specifically focused on craniofacial etiology.
